# Hunters Searching among Starry Nights and at the Edges of Life

**DOI:** 10.3201/eid2601.AC2601

**Published:** 2020-01

**Authors:** Byron Breedlove

**Affiliations:** Centers for Disease Control and Prevention, Atlanta, Georgia, USA

**Keywords:** art science connection, emerging infectious diseases, art and medicine, about the cover, Charles Burchfield, Orion in December, Hunters Searching among Starry Nights and at the Edges of Life, viruses, astronomy, astrovirology, Orion

**Figure:**
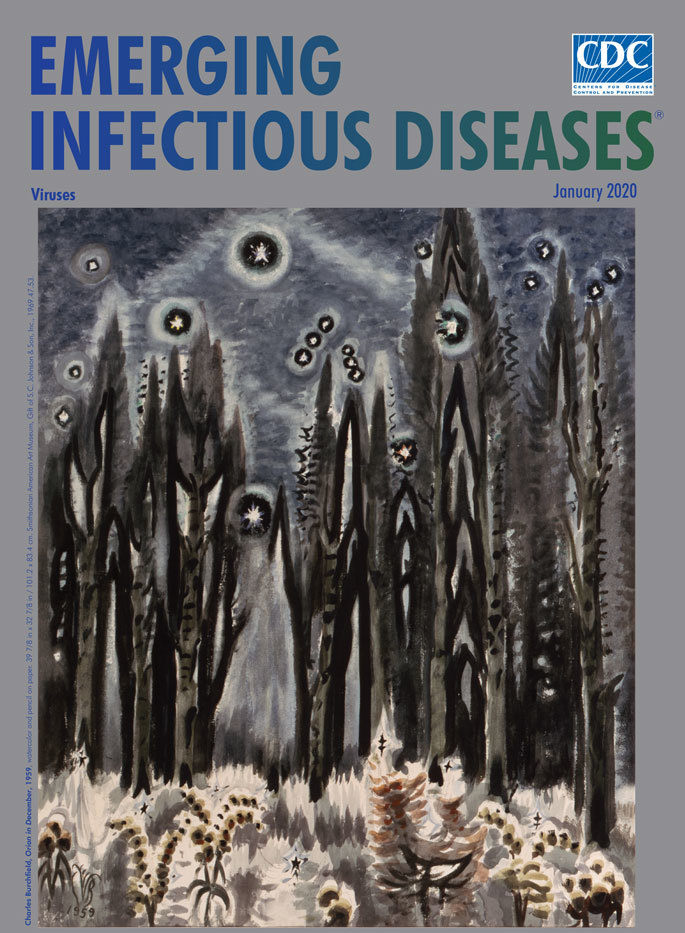
**Charles Burchfield, *Orion in December* 1959**. Watercolor and pencil on paper. 39 7/8 in × 32 7/8 in/101.2 × 83.4 cm. Smithsonian American Art Museum, Gift of S.C. Johnson & Son, Inc., 1969.47.53.

Astronomers and astrophysicists work with immense, mind-boggling numbers. Caleb A. Scharf, Director of Astrobiology at Columbia University, writes “On a finite world a cosmic perspective isn’t a luxury, it’s a necessity.” Consider that the distance between the earth and sun equals ≈100 million miles. One of the nearest stars to Earth, Alpha Centauri shimmers some 4.4 light-years away. Put another way, if the distance from the earth to the sun were fixed at 1 inch, then one of Earth’s closest neighbors would be 4.4 miles (7 km) distant. The Milky Way, our home galaxy, comprises something in the range of 200–300 billion stars. It is nestled among the other 2 trillion or so galaxies estimated to populate the observable universe, postulated to have a radius of 13.8 billion light-years.

However, the figure ascribed to the quantity of terrestrial viruses, an estimated 10^31^, eclipses even those immense numbers. That means there are potentially 5–10 million times more viruses on Earth than stars thought to exist in the observable universe. Science writer Carl Zimmer offers this perspective: “If you were to stack one virus on top of another, you’d create a tower that would stretch beyond the moon, beyond the sun, beyond Alpha Centauri, out past the edge of the Milky Way, past neighboring galaxies, to reach a height of 200 million light years.”

Viruses, which have no cell nucleus and consist of DNA or RNA enveloped in a protein, are not classified among the 5 kingdoms of living things: bacteria, fungi, protists, plants, and animals. Debate lingers about whether viruses are alive. Virologists Marc H.V. van Regenmortel and Brian W.J. Mahy explain that “A virus becomes part of a living system only after it has infected a host cell and its genome becomes integrated with that of the cell. Viruses are replicated only through the metabolic activities of infected cells, and they occupy a unique position in biology. They are nonliving infectious entities that can be said, at best, to lead a kind of borrowed life.” 

Viruses exist at the edge of life and do not themselves exhibit life functions. They replicate and mutate efficiently and quickly, enabling them to readily adapt to new hosts. Some viruses can survive in the most extreme conditions. Their vast, diverse population outnumbers other cellular life-forms by at least 10-fold. Viruses have played a crucial role in the evolution and adaptability of life, and although viruses cause many human illnesses, most do not cause disease and death. 

In the past few decades, emerging infectious diseases researchers have identified a number of deadly viruses that infect humans. Recently discovered viruses include dengue viruses, Ebola virus, Marburg virus, many hantavirus, HIV, influenza viruses, rotaviruses, Nipah virus, and Middle East respiratory syndrome coronavirus.

Viruses also cause myriad diseases in animals, including rabies, foot-and-mouth disease, bluetongue, avian influenza, peste des petits ruminants, and swine flu. A number of diseases in plants, such as various leaf roll and leaf curl diseases, mosaic diseases, and ring spot, are caused by viruses. The bottom line is that viruses infect organisms at every scale, from bacteria to blue whales.

As virology enters its second century, some researchers are starting to investigate whether viruses can survive extraterrestrially. Berliner, Mochizuki, and Stedman, authors of a review article in Astrobiology, lay out some short-term and long-term priorities for astrovirology research and discuss difficulties and stratagems for finding viral biosignatures on Earth and in extraterrestrial environments. As they point out, anywhere life exists on Earth, viruses also exist in abundance, and they note “we do need to learn more about viruses on modern Earth before we look elsewhere, but let’s start looking.” 

*Orion in December*, this month’s cover art, connects the realms of Earth and space and invokes Orion the Hunter from Greek mythology. Its creator, Charles Burchfield may not be the most recognized name in the canon of 20th century artists but is some considered by some a classic example of the “artist’s artist.” (See Military Magic or Nature’s Fool, the cover essay for the April 2012 EID, for more details about Burchfield.) He favored watercolors, often combined with gouache, pencil, charcoal, or pastels. Art scholar Paloma Alarcó notes that “Burchfield, who was well versed in astronomy, felt a special attraction for the sky and heavenly bodies—not only the sun and moon but also the Pleiades and certain constellations of stars, particularly Orion, which appears in winter.” Indeed, the artist featured Orion, one most prominent and recognizable constellations visible from almost everywhere on earth, in 3 other paintings.

Orion’s bright stars, including the supergiants Betelgeuse and Rigel, shimmer so brightly they seem to vibrate. The constellation fills the winter sky and dips into the trees, cinching together earth and sky. Burchfield links the points of lights within Orion using thick, hazy lines, ensuring viewers cannot miss the overall form. The spire-shaped trees stand like rockets poised to launch into the night sky. In the foreground, the underbrush, heavy with frost and ice, reaches skyward and seems to crackle like pale flames lapping at the tree trunks. 

Heighted hallucinatory effects such as these were integral to Burchfield’s approach in the late stages of his career. The American Art at the Phillips Collection notes, “Burchfield’s subjects are unsophisticated but gain immediacy through energetic two-dimensional patterns that animate the surface of his pictures and evoke sensations of the subject’s particular play of light, weather conditions, and even sound.” 

Orion was a hunter in Greek mythology, and the constellation Burchfield painted happens to be a focal point in the hunt for exoplanets, some of which may have conditions favorable for viruses and by association the potential for extraterrestrial life. Any such discoveries would further amplify the already gargantuan numbers associated with astronomy and virology.
